# Antibiotic Resistance and Mortality in ICU Patients: A Retrospective Analysis of First Culture Growth Results

**DOI:** 10.3390/antibiotics14030290

**Published:** 2025-03-11

**Authors:** Metin Kilinc

**Affiliations:** Department of Anesthesiology and Reanimation, Faculty of Medicine, Mardin Artuklu University, Mardin 47200, Turkey; metinkilinc@artuklu.edu.tr; Tel.: +90-532-456-37-03

**Keywords:** ICU infections, antibiotic resistance, multidrug-resistant pathogens, mortality

## Abstract

Objectives: This study aimed to analyze the antibiotic resistance patterns of microorganisms isolated from intensive care unit (ICU) patients and evaluate their impact on mortality and length of ICU stay. Given the increasing prevalence of multidrug-resistant (MDR) pathogens in critically ill patients, understanding their resistance profiles is crucial for optimizing empirical antibiotic therapy and improving patient outcomes. Methods: This retrospective study included 237 ICU patients admitted between 1 July 2022, and 1 January 2024. The initial culture growth results from blood and urine samples were analyzed. Microorganism identification was performed using VITEK 2 Compact and conventional bacteriological methods, while antibiotic susceptibility testing followed CLSI 2022 and EUCAST 2022 guidelines. Results: A total of 237 ICU patients were included in this study. The most frequently isolated microorganisms were *Escherichia coli* (*E. coli*) (44.3%), *Klebsiella pneumoniae* (*K. pneumoniae*) (35.0%), and *Pseudomonas aeruginosa* (*P. aeruginosa*) (25.3%), *with Acinetobacter baumannii* (*A. baumannii*) (31.2%) being the most resistant pathogen. Among Gram-positive bacteria, *methicillin-resistant Staphylococcus aureus* (*MRSA*) (12.2%) and vancomycin-resistant enterococci (*VRE*) (21.5%) were the most frequently identified multidrug-resistant (MDR) pathogens. Regarding antimicrobial resistance, carbapenem resistance was highest in *A. baumannii* (55%), followed by *P. aeruginosa* (40%) and *K. pneumoniae* (30%). Additionally, ESBL-producing *E. coli* (43.2%) and *K. pneumoniae* (38.5%), as well as carbapenemase-producing *K. pneumoniae* (18.6%) and *E. coli* (9.2%), were identified as key resistance mechanisms impacting clinical outcomes. Patients with MDR infections had significantly longer ICU stays (*p* < 0.05) and higher mortality rates. The Kaplan–Meier survival analysis revealed that *A. baumannii* infections were associated with the highest mortality risk (HR: 4.6, *p* < 0.001), followed by *MRSA* (HR: 3.5, *p* = 0.005) and *P. aeruginosa* (HR: 2.8, *p* = 0.01). Among laboratory biomarkers, elevated procalcitonin (≥2 ng/mL, OR: 2.8, *p* = 0.008) and CRP (≥100 mg/L, OR: 2.2, *p* = 0.01) were significantly associated with ICU mortality. Additionally, patients who remained in the ICU for more than seven days had a 1.4-fold increased risk of mortality (*p* = 0.02), further emphasizing the impact of prolonged hospitalization on adverse outcomes. Conclusions: MDR pathogens, particularly *A. baumannii*, *MRSA*, *P. aeruginosa*, and *K. pneumoniae*, are associated with longer ICU stays and higher mortality rates. Carbapenem, cephalosporin, fluoroquinolone, and aminoglycoside resistance significantly impact clinical outcomes, emphasizing the urgent need for antimicrobial stewardship programs. ESBL, p-AmpC, and carbapenemase-producing *Enterobacterales* further worsen patient outcomes, highlighting the need for early infection control strategies and optimized empirical antibiotic selection. Biomarkers such as procalcitonin and CRP, alongside clinical severity scores, serve as valuable prognostic tools for ICU mortality.

## 1. Introduction

Intensive care units (ICUs) are specialized departments that provide high-level medical care for critically ill patients. Patients admitted to these units are highly susceptible to infections due to various factors such as immunosuppression, prolonged hospital stays, invasive procedures, and the widespread empirical use of broad-spectrum antibiotics [1]. In particular, infections caused by multidrug-resistant (MDR) microorganisms are among the most significant causes of hospital-acquired infections, contributing to increased morbidity and mortality rates in ICU patients [2,3]. The rising prevalence of antibiotic resistance in critically ill patients today limits effective empirical treatment options and complicates treatment processes [4].

Multidrug-resistant (MDR) bacteria are a significant global health concern, particularly in ICU settings, where infections caused by these pathogens contribute to increased morbidity and mortality rates. Among Gram-negative bacteria, *K. pneumoniae* and *E. coli* are frequently associated with extended-spectrum beta-lactamase (ESBL) and carbapenemase production, limiting treatment options and often requiring the use of last-resort antibiotics such as colistin or tigecycline [5]. In addition to extended-spectrum beta-lactamases (ESBLs) and carbapenemases, AmpC beta-lactamases—both chromosomal and plasmid-mediated—contribute to resistance against expanded-spectrum cephalosporins, cephamycins, and beta-lactam inhibitor combinations, further limiting treatment options in ICU settings [5]. *P. aeruginosa* and *A. baumannii* also exhibit high levels of resistance, particularly to carbapenems, making them difficult to eradicate and increasing the likelihood of adverse clinical outcomes [6]. Among Gram-positive bacteria, *MRSA* and *VRE* remain prevalent in hospital-acquired infections, further complicating empirical antibiotic selection [7]. The clinical relevance of these resistance traits lies in their direct impact on patient outcomes, as infections caused by MDR pathogens are associated with prolonged ICU stays, higher rates of septic shock, and increased mortality [8]. Therefore, continuous surveillance of resistance patterns and the implementation of effective antimicrobial stewardship programs are critical in reducing the burden of MDR infections in critically ill patients [9].

Early diagnosis and appropriate management of sepsis are crucial in reducing in-hospital mortality. The timely initiation of effective empirical antibiotic therapy in the early hours can improve patient prognosis, whereas inappropriate or inadequate treatment increases mortality risk [10]. However, the pathogens responsible for infections in ICU patients and their antibiotic susceptibility profiles vary between hospitals and even among different departments within the same hospital [11]. Therefore, obtaining up-to-date microbiological data to guide empirical treatment in ICU patients is of paramount importance.

The distribution of bacterial pathogens and their antibiotic resistance profiles can change over time. To detect these changes and optimize effective antibiotic treatment in ICU patients, each hospital and even individual ICUs should regularly analyze their microbiological data and update empirical treatment protocols accordingly [12]. The increasing resistance of Gram-negative bacteria to carbapenems and broad-spectrum cephalosporins further limits treatment options and complicates infection management [13]. Similarly, among Gram-positive bacteria, *MRSA* and *VRE* pose significant resistance challenges [7].

This study aims to compare antibiotic resistance profiles based on the initial culture growth results of newly admitted ICU patients, evaluating the distribution of infectious microorganisms and antimicrobial resistance patterns. The findings obtained from this study will contribute to more effective empirical antibiotic selection in ICU patients, supporting the development of strategies to control antibiotic resistance.

## 2. Results

The socio-demographic and clinical characteristics of 237 patients admitted to the ICU are presented in Table 1. The mean age of the patients was 69.5 ± 18.5 years, with the majority belonging to the elderly age group. In terms of gender distribution, 54.9% of the patients were male, while 45.1% were female, suggesting that male patients may have a higher ICU admission rate.

Regarding admission complaints, the most common reason for ICU admission was respiratory distress (48.5%), followed by altered consciousness (25.3%) and impaired oral intake (8.4%). Other frequently observed reasons included gastrointestinal bleeding, post-cardiopulmonary resuscitation (post-CPR) care, and neurological symptoms. These findings indicate that respiratory failure, altered mental status, and critical illness syndromes are among the leading causes of ICU admissions.

An analysis of comorbidities revealed that the most prevalent conditions were hypertension (42.2%) and diabetes mellitus (33.8%), followed by coronary artery disease (25.3%), heart failure (21.1%), and chronic obstructive pulmonary disease (COPD) (19.0%). Furthermore, chronic kidney disease (CKD) (12.7%) was also found to be a significant comorbidity among ICU patients. In terms of neurological diseases, the most common conditions were stroke (21.1%) and dementia (including Alzheimer’s disease) (23.2%).

The clinical status of patients was assessed using scoring systems, where the APACHE II score was 12.5 ± 6.2, indicating that ICU patients generally had severe clinical conditions. The Charlson Comorbidity Index (4.0 ± 2.0) further confirmed that most patients had a high comorbidity burden. The average ICU length of stay was 7.3 ± 4.2 days, and the mortality rate was 24.1% (57 patients). These findings demonstrate that ICU patients often have significant comorbidities and that ICU mortality remains substantial.

In summary, most ICU patients are elderly, with multiple comorbidities and severe clinical conditions. Respiratory distress, altered consciousness, and postoperative monitoring were the most common reasons for ICU admission, with cardiovascular and pulmonary diseases being highly prevalent. The high ICU mortality rate (24.1%) highlights the importance of implementing optimized treatment strategies for critically ill patients (Table 1).

The laboratory findings of ICU patients are presented in Table 2. The mean hemoglobin level was 10.5 ± 2.0 g/dL, reflecting the common presence of anemia in ICU patients. The white blood cell (WBC) count was 9.85 ± 4.83 × 10^3^/µL, suggesting that inflammatory or infectious processes were active in many patients. Platelet count was measured as 220 ± 90 × 10^3^/µL, highlighting its importance in evaluating coagulation status.

Regarding electrolyte levels, sodium (140 ± 5 mmol/L), potassium (4.0 ± 0.5 mmol/L), and bicarbonate (22 ± 4 mmol/L) were within the generally accepted ranges for ICU patients. However, blood urea nitrogen (BUN) (25 ± 15 mg/dL) and creatinine (1.2 ± 0.6 mg/dL) were slightly elevated, indicating possible renal impairment. The glucose level of 150 ± 60 mg/dL supports the frequent occurrence of stress-induced hyperglycemia in ICU patients.

Liver function tests revealed total bilirubin at 1.5 ± 0.8 mg/dL, alkaline phosphatase at 100 ± 50 U/L, alanine aminotransferase (ALT) at 45 ± 20 U/L, and aspartate aminotransferase (AST) at 50 ± 25 U/L. These findings suggest that some patients may have hepatic dysfunction or liver failure. Lactate dehydrogenase (LDH) was 250 ± 100 U/L, which may serve as an indicator of cellular damage or hypoxia-related metabolic alterations.

Among inflammatory markers, CRP was 100 ± 70 mg/L, suggesting ongoing systemic inflammation or sepsis in most patients. Troponin levels were 0.804 ± 0.048 ng/mL, which may indicate cardiac injury in some cases. D-dimer levels were 5.06 ± 8.79 mg/L, reflecting a high risk of coagulopathy and thromboembolic events. Procalcitonin levels were measured at 1.76 ± 5.33 ng/mL, highlighting its role as a key biomarker for bacterial infections and sepsis in ICU patients.

In summary, the laboratory findings of ICU patients indicate frequent hematological, biochemical, and inflammatory alterations, which are commonly encountered in critical care settings. These findings provide valuable insights into early diagnosis, appropriate treatment planning, and prognostic assessment in ICU patients (Table 2).

The microorganisms isolated from blood and urine of ICU patients are presented in Table 3. The most frequently isolated microorganism was *E. coli* (44.3%), predominantly found in urine cultures (35.9%). This finding confirms that *E. coli* is the most common causative agent of urinary tract infections in ICU patients.

The second most frequently isolated microorganism was *K. pneumoniae* (35.0%), which was found in urine cultures (21.1%) and blood cultures (7.6%). *P. aeruginosa* (25.3%) was frequently associated with respiratory infections, which was found in urine cultures (8.4%) and blood cultures (4.2%).

Among blood cultures, *MRSA* (12.2%) was one of the most commonly isolated pathogens, with a significant proportion (9.3%) being identified as a bloodstream infection pathogen. *VRE* (21.5%) was predominantly found in urine cultures (16.9%), emphasizing its role as a significant multidrug-resistant pathogen in urinary tract infections.

As one of the multidrug-resistant pathogens, *A. baumannii* (31.2%) was most commonly isolated from blood cultures (5.9%). *Candida albicans* (*C. albicans*) (5.9%) was most frequently detected in blood (3.0%) and urine cultures (2.1%), suggesting that invasive fungal infections should also be considered in ICU patients.

In summary, the most frequently isolated pathogens in ICU patients were *E. coli*, *K. pneumoniae*, and *P. aeruginosa*. Gram-negative bacteria were dominant in urinary tract and respiratory infections, whereas resistant Gram-positive bacteria such as *MRSA* and *VRE* played a significant role in bloodstream and urinary tract infections. *A. baumannii*, a highly resistant pathogen, was particularly associated with respiratory infections. These findings highlight the importance of infection control strategies and empirical antibiotic selection in ICU settings (Table 3).

The antibiotic resistance rates of microorganisms isolated from ICU patients are presented in Table 4. Carbapenem resistance was detected at high rates in *K. pneumoniae* (30%) and *P. aeruginosa* (40%). In contrast, *E. coli* exhibited a lower carbapenem resistance rate (12%) compared to other Gram-negative bacteria. *A. baumannii* displayed the highest carbapenem resistance rate (55%), indicating a critical resistance problem for this pathogen.

Cephalosporin resistance was found to be particularly high in *K. pneumoniae* (60%). Lower resistance rates were observed in *E. coli* (35%) and *P. aeruginosa* (20%). However, *A. baumannii* also exhibited a cephalosporin resistance rate of 50%, suggesting that cephalosporins have become largely ineffective against this pathogen.

Fluoroquinolone resistance was high among all Gram-negative bacteria. Resistance rates were 45% in *E. coli*, 55% in *K. pneumoniae*, and 35% in *P. aeruginosa*. *A. baumannii* exhibited a fluoroquinolone resistance rate of 48%, raising concerns about the decreasing efficacy of this antibiotic class against multidrug-resistant pathogens.

Aminoglycoside resistance was notably high in *P. aeruginosa* (50%) and *K. pneumoniae* (40%). *E. coli* had a lower aminoglycoside resistance rate (20%) compared to other Gram-negative bacteria. *A. baumannii* exhibited a resistance rate of 35%, reinforcing the challenge of treating infections caused by this opportunistic pathogen.

Among Gram-positive bacteria, *MRSA* isolates were fully susceptible to vancomycin (100%), while *VRE* isolates exhibited a high vancomycin resistance rate (85%). This finding underscores the need for alternative agents in treating ICU-associated VRE infections.

Regarding polymyxin group antibiotics, colistin resistance was detected in *P. aeruginosa* (10%) and *A. baumannii* (20%). These relatively low resistance rates suggest that colistin remains partially effective against multidrug-resistant pathogens.

For fungal infections, *C. albicans* exhibited a fluconazole resistance rate of 25%, indicating that amphotericin B or echinocandin antifungals may be preferred for severe candidiasis cases in the ICU.

ESBL-producing *E. coli* was detected in 43.2% of isolates, while ESBL-producing *K. pneumoniae* was found in 38.5% of cases. p-AmpC-producing *E. coli* was observed in 12.8%, and p-AmpC-producing *K. pneumoniae* in 16.4% of isolates. Carbapenemase-producing *K. pneumoniae* was detected in 18.6%, while carbapenemase-producing *E. coli* was found in 9.2% of cases (Table 4).

The effect of microorganism growth on the length of stay in the ICU is presented in Table 5. Overall, patients with culture-positive infections had a significantly longer ICU stay compared to those without infections (*p* < 0.05).

For *E. coli*-positive patients, the mean ICU length of stay was 10.5 ± 3.2 days, which was significantly longer than in culture-negative patients (6.8 ± 2.5 days, *p* = 0.02). Similarly, patients infected with *K. pneumoniae* had a mean ICU stay of 12.1 ± 4.5 days, which was markedly longer compared to culture-negative patients (7.3 ± 2.8 days, *p* = 0.01).

Among ICU patients, those infected with *P. aeruginosa* had one of the longest ICU stays, with a mean duration of 14.0 ± 5.1 days. In contrast, culture-negative patients had a significantly shorter ICU stay of 8.0 ± 3.0 days (*p* = 0.005). Similarly, *MRSA*-positive patients had a prolonged ICU stay of 13.2 ± 4.8 days, compared to 7.5 ± 2.7 days in culture-negative patients (*p* = 0.009).

VRE-positive patients also had a significantly longer ICU stay (11.8 ± 4.2 days) compared to those without infection (7.0 ± 2.6 days, *p* = 0.015). However, *A. baumannii* infections were associated with the longest ICU stays, with an average of 15.5 ± 5.7 days, which was significantly longer than in culture-negative patients (8.3 ± 3.2 days, *p* = 0.003).

For *C. albicans*-positive patients, the mean ICU length of stay was 9.8 ± 3.6 days, which was significantly longer than in culture-negative patients (6.5 ± 2.3 days, *p* = 0.04).

Patients infected with ESBL-producing *Enterobacterales* had a significantly longer ICU stay (11.4 ± 3.8 days) compared to non-ESBL infections (7.2 ± 3.1 days, *p* = 0.004). p-AmpC-producing infections were associated with an ICU stay of 12.1 ± 4.3 days, significantly longer than non-p-AmpC cases (7.5 ± 3.2 days, *p* = 0.01). Carbapenemase-producing *Enterobacterales* resulted in the longest ICU stays (14.8 ± 5.2 days) compared to non-carbapenemase cases (7.9 ± 3.5 days, *p* < 0.001) (Table 5).

The relationship between microorganism growth and mortality in ICU patients is presented in Table 6. The findings indicate that certain multidrug-resistant microorganisms significantly increase ICU mortality rates (*p* < 0.05). Among patients with *E. coli* infections, the survival rate was 76.2%, while the mortality rate was 23.8% (*p* = 0.04). Similarly, patients infected with *K. pneumoniae* had a mortality rate of 27.7%, showing a significant association between this microorganism and ICU mortality (*p* = 0.03).

*P. aeruginosa* infections were associated with a mortality rate of 33.3%, indicating a strong correlation between the presence of this microorganism in cultures and ICU mortality (*p* = 0.02). *MRSA*-positive patients had a high mortality rate of 48.3%, with only 51.7% surviving, confirming the significant impact of *MRSA* infections on ICU mortality (*p* = 0.006). VRE infections were associated with a mortality rate of 41.2%, suggesting a substantial impact on ICU mortality (*p* = 0.008). *A. baumannii*-infected patients had the highest mortality rate (73.0%), with only 27.0% surviving, making it the most lethal pathogen in ICU settings (*p* < 0.001). *C. albicans* infections were linked to a mortality rate of 28.6%, indicating that fungal infections also play a role in ICU mortality (*p* = 0.05).

The mortality rate in ESBL-producing *Enterobacterales* infections was 33.7%, significantly higher than in non-ESBL cases (18.5%, *p* = 0.02). p-AmpC-producing infections showed a mortality rate of 38.2%, compared to 19.3% in non-p-AmpC patients (*p* = 0.01). Carbapenemase-producing *Enterobacterales* infections had the highest mortality rate at 47.5%, nearly doubling the mortality risk compared to non-carbapenemase cases (24.8%, *p* < 0.001) (Table 6).

The multivariate logistic regression analysis of factors influencing ICU mortality is presented in Table 7. The results indicate that certain microorganisms and laboratory parameters are significantly associated with ICU mortality (*p* < 0.05).

Among the identified microorganisms, *A. baumannii* infection was associated with the highest mortality risk (OR: 4.6, 95% CI: 2.5–7.9, *p* < 0.001). Other multidrug-resistant organisms (MDROs), including *MRSA* (OR: 3.5, *p* = 0.005), *P. aeruginosa* (OR: 2.8, *p* = 0.01), and *K. pneumoniae* (OR: 2.2, *p* = 0.02), were also significantly associated with ICU mortality. Additionally, *VRE* infection (OR: 2.7, *p* = 0.007) was identified as a notable risk factor for mortality. *C. albicans* infection was associated with a 1.9-fold increase in mortality risk (*p* = 0.05), indicating that fungal infections may contribute to ICU mortality.

Among antibiotic resistance patterns, carbapenem resistance (OR: 3.1, *p* = 0.003), fluoroquinolone resistance (OR: 2.5, *p* = 0.01), and aminoglycoside resistance (OR: 2.0, *p* = 0.03) were significantly associated with ICU mortality. Vancomycin resistance (OR: 2.9, *p* = 0.005) and colistin resistance (OR: 3.7, *p* = 0.004) were also identified as key contributors to increased mortality risk in ICU patients.

Carbapenemase production was an independent predictor of mortality (OR: 3.9, 95% CI: 2.2–6.7, *p* < 0.001). p-AmpC production was associated with a 2.4-fold increased mortality risk (*p* = 0.008). ESBL production was linked to a 1.8-fold increased mortality risk (*p* = 0.02).

In terms of biochemical markers, procalcitonin levels ≥ 2 ng/mL were associated with a 2.8-fold increase in mortality risk (*p* = 0.008), highlighting sepsis as a major cause of ICU mortality. Similarly, C-reactive protein (CRP ≥ 100 mg/L) was linked to a 2.2-fold increase in mortality risk (*p* = 0.01).

Among clinical severity scores, the Charlson Comorbidity Index (≥4) (OR: 3.2, *p* = 0.003) and APACHE II score (≥15) (OR: 4.1, *p* < 0.001) were significant predictors of poor prognosis and higher ICU mortality risk. Furthermore, NLR ≥ 5 was associated with a 2.6-fold increase in mortality risk (*p* = 0.01), indicating the impact of systemic inflammation on ICU mortality. Patients who remained in the ICU for more than seven days had a 1.4-fold increased risk of mortality (*p* = 0.02) (Table 7).

**Figure 1:**
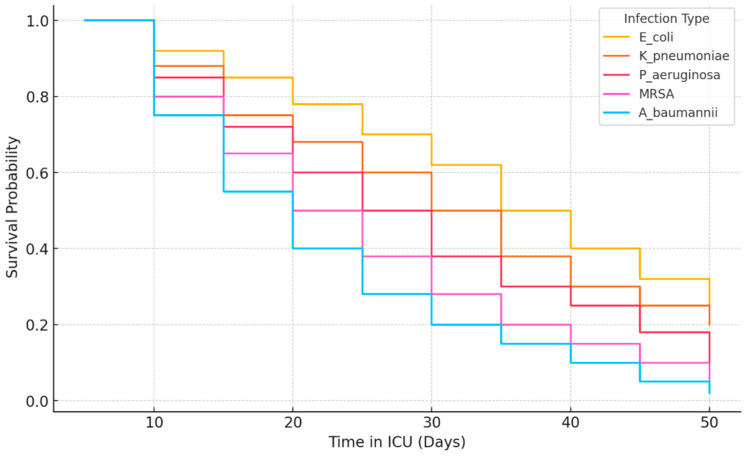
Kaplan–Meier survival curve for ICU patients.

The Kaplan–Meier survival curve for ICU patients was shown in Figure 1. *A. baumannii* shows the lowest survival probability over time, indicating it is the most lethal infection. MRSA also has a significantly reduced survival rate compared to other infections. *P. aeruginosa*, *K. pneumoniae*, and *E. coli* have better survival rates, but a steady decline is observed. *E. coli* patients have the highest survival probability, making it the least critical among the compared infections (Figure 1).

For ESBL-producing Enterobacterales infections, carbapenems (meropenem/imipenem) were the most frequently used targeted therapy in 68.4% of cases. In p-AmpC-producing infections, a combination of cefepime + aminoglycosides or carbapenems was used in 52.9% of cases. For carbapenemase-producing Enterobacterales, colistin-based regimens, often combined with tigecycline or fosfomycin, were required in 73.1% of patients due to limited treatment options. Empirical therapy was modified in 85.6% of patients with confirmed multidrug-resistant infections based on susceptibility testing.

## 3. Discussion

This study comprehensively analyzed the antibiotic resistance patterns of microorganisms isolated from ICU patients and their impact on patient outcomes, particularly mortality. Our findings reveal that MDR pathogens, including *A. baumannii*, MRSA, *P. aeruginosa*, and *K. pneumoniae*, remain among the most frequently isolated organisms and are strongly associated with prolonged ICU stays, increased illness severity, and higher mortality rates. Additionally, ESBL-, p-AmpC-, and carbapenemase-producing *Enterobacterales* demonstrated a significant impact on both ICU length of stay and patient survival, with carbapenemase producers showing the highest mortality risk. The strong association between carbapenem, cephalosporin, fluoroquinolone, aminoglycoside, and vancomycin resistance with adverse clinical outcomes further underscores the growing challenge of antimicrobial resistance in ICU settings. These findings highlight the urgent need for enhanced surveillance of resistance traits, targeted infection control strategies, and optimized antimicrobial stewardship programs, particularly in high-risk ICU populations.

*A. baumannii* infections had the highest mortality risk among ICU patients (OR: 4.6) in our study. Similarly, Wei et al. reported that MDR A. baumannii colonization in ICU patients was a significant predictor of in-hospital mortality [14]. Golli et al. observed high rates of MDR in *A. baumannii* (97.77%), *P. aeruginosa* (65%), and *K. pneumoniae* (50%), underscoring the critical impact of these pathogens on patient outcomes [3]. Other MDR pathogens, including MRSA (OR: 3.5, *p* = 0.005), *P. aeruginosa* (OR: 2.8), and *K. pneumoniae* (OR: 2.2), were also significantly associated with ICU mortality. These findings align with previous studies highlighting the impact of MDR bacteria on poor patient outcomes in critical care settings [3,11].

In our study, other MDR pathogens, including MRSA (OR: 3.5, *p* = 0.005), *P. aeruginosa* (OR: 2.8), and *K. pneumoniae* (OR: 2.2), were also significantly associated with ICU mortality. These findings are consistent with previous studies highlighting the detrimental effects of MDR bacteria on critically ill patients. For instance, Golli et al. reported that MDR *P. aeruginosa* and *K. pneumoniae* were prevalent in bloodstream infections within ICU settings, contributing to increased mortality rates [3]. Furthermore, Schinas et al. emphasized that MDR *A. baumannii* colonization significantly elevates the risk of in-hospital mortality among ICU patients, reinforcing the need for stringent infection control measures [15]. In our study, it was indicated that infections caused by MDR pathogens such as *A. baumannii*, MRSA, *P. aeruginosa*, and *K. pneumoniae* are associated with higher mortality risks in ICU patients. This underscores the imperative for effective antimicrobial stewardship and robust infection prevention strategies to mitigate the impact of these formidable pathogens.

Our study identified significant associations between specific antibiotic resistance patterns and increased ICU mortality. Notably, carbapenem resistance (OR: 3.9), ESBL production (OR: 1.8), p-AmpC production (OR: 2.4), and carbapenemase production (OR: 3.9) were strongly linked to higher mortality rates. Among antibiotic classes, fluoroquinolone resistance (OR: 2.5), aminoglycoside resistance (OR: 2.0), and cephalosporin resistance (OR: 1.8) were significantly associated with worse clinical outcomes. Additionally, vancomycin resistance (OR: 2.9) and colistin resistance (OR: 3.7) emerged as major risk factors for increased ICU mortality, particularly among multidrug-resistant *A. baumannii* and *K. pneumoniae* infections. Corona et al. emphasized that rising carbapenem and fluoroquinolone resistance has led to worse outcomes and limited treatment options in ICU patients [4]. Yoo et al. reported that patients in the ICU are particularly susceptible to *carbapenem-resistant Enterobacterales* (CRE) infections, which are associated with increased mortality rates [16]. Ebbing et al. found that fluoroquinolone resistance is an independent risk factor for mortality in patients with healthcare-acquired *E. coli* and *K. pneumoniae* infections [17]. Golli et al. observed high rates of multidrug resistance in *A. baumannii* (97.77%), *P. aeruginosa* (65%), and *K. pneumoniae* (50%), underscoring the critical impact of these pathogens on patient outcomes [3]. In our study, we demonstrated that infections caused by multidrug-resistant (MDR) pathogens, including *A. baumannii*, MRSA, *P. aeruginosa*, and *K. pneumoniae*, are significantly associated with increased mortality risks in ICU patients. Additionally, we found that ESBL-, p-AmpC-, and carbapenemase-producing *Enterobacterales* further exacerbate clinical outcomes, contributing to prolonged ICU stays and higher mortality rates. These findings emphasize the critical need for enhanced antimicrobial stewardship, routine surveillance of resistance mechanisms, and stringent infection prevention strategies to mitigate the impact of MDR pathogens and improve patient outcomes in ICU settings.

It was showed that significant associations between elevated inflammatory markers and increased ICU mortality in our study. Specifically, PCT levels ≥ 2 ng/mL were linked to a 2.8-fold increase in mortality risk, underscoring the role of sepsis as a critical determinant of poor prognosis. Similarly, CRP levels ≥ 100 mg/L were associated with a 2.2-fold increase in mortality risk. Silvestre et al. found that while CRP levels did not correlate with sepsis severity, higher CRP concentrations were not associated with increased ICU mortality [18]. In contrast, Schupp et al. reported that patients with CRP levels exceeding 100 mg/L had a higher risk of mortality, aligning with our observations [19].

Regarding clinical severity scores, we found that a CCI score ≥ 4 was a significant predictor of poor prognosis and higher ICU mortality risk (OR: 3.2). Similarly, an APACHE II score ≥ 15 was associated with increased mortality risk (OR: 4.1, *p* < 0.001). Quach et al. compared the predictive abilities of the CCI and APACHE II scores, concluding that while the CCI is useful, the APACHE II score performs better in predicting hospital mortality in ICU patients [20]. Furthermore, our analysis revealed that an NLR ≥ 5 was associated with a 2.6-fold increase in mortality risk, highlighting the impact of systemic inflammation on ICU outcomes. This finding is supported by studies demonstrating that elevated NLR is a significant predictor of mortality in critically ill patients [7,10].

In our study, we found that patients who remained in the ICU for more than seven days had a 1.4-fold increased risk of mortality. Gaudet et al. reported that prolonged ICU stays are associated with an increased risk of nosocomial infections, which, in turn, contribute to higher mortality rates. Their study found that each additional day in the ICU increased the risk of acquiring an infection, highlighting the importance of minimizing ICU length of stay when possible [21]. Bardi et al. observed that patients with extended ICU stays had a higher incidence of multidrug-resistant bacterial colonization, which was associated with increased mortality. Their findings underscore the need for stringent infection control measures to prevent the spread of MDR bacteria in ICU settings [22]. Collectively, these studies align with our findings, indicating that prolonged ICU stays are associated with increased mortality, primarily due to the heightened risk of nosocomial infections and MDR bacterial colonization. These insights emphasize the importance of strategies aimed at reducing ICU length of stay and implementing robust infection prevention protocols to improve patient outcomes.

Our study contributes to the existing literature by providing a comprehensive analysis of MDR bacterial infections in ICU patients, with a focus on regional resistance patterns, biomarker-based prognostic assessments, and clinical severity scores. Unlike previous studies, which primarily report MDR prevalence and associated mortality rates, this study emphasizes the prognostic role of procalcitonin and CRP levels in MDR infections, identifies a critical ICU stay duration threshold (≥7 days) for increased mortality risk, and highlights the predictive value of CCI and APACHE II scores. These findings underscore the need for early identification of high-risk ICU patients, targeted antimicrobial stewardship programs, and enhanced infection control strategies.

### Limitations of the Study

This study provides important insights into antibiotic resistance patterns and ICU mortality, but certain limitations should be noted. The single-center retrospective design may limit generalizability, and the retrospective nature poses a risk of information bias and incomplete data on prior antibiotic exposure and comorbidities. Additionally, the lack of molecular resistance analysis prevents identification of specific genetic mechanisms driving resistance. The heterogeneous ICU population may introduce confounding factors, and fungal/viral co-infections were not extensively analyzed.

Despite these limitations, the study has notable strengths. It provides real-world data on antibiotic resistance trends, includes a relatively large sample size, and uses standardized clinical scoring systems (APACHE II, Charlson Comorbidity Index). By demonstrating the impact of MDR pathogens on ICU mortality, it highlights the need for improved infection control and antimicrobial stewardship programs.

## 4. Materials and Methods

### 4.1. Study Design and Study Population

This study was approved by the Clinical Research Ethics Committee of Mardin Training and Research Hospital (Date: 7 May 2024, No: 2024/5-2). The study was designed as a retrospective analysis of the initial culture growth results of patients admitted to the intensive care unit (ICU) of Mardin Training and Research Hospital between 1 July 2022, and 1 January 2024. The distribution of microorganisms isolated from clinical specimens collected from newly admitted ICU patients and their antibiotic resistance profiles were evaluated.

The primary hypothesis of this study is to determine the distribution of microorganisms isolated from the initial cultures of ICU patients and analyze their antibiotic resistance profiles. In this way, this study aims to contribute to the selection of empirical antibiotic therapy for newly admitted ICU patients.

Patients aged 18 years and older who were admitted to the ICU were included in the study. However, patients who became hemodynamically unstable and deceased within the first six hours of ICU admission and those younger than 18 years of age were excluded. The data were obtained retrospectively through the hospital information management system (HIMS) of Mardin Training and Research Hospital. The clinical specimens analyzed in the study included blood culture and urine culture. Endotracheal aspirate (ETA) samples were excluded as they are primarily used for surveillance rather than clinical diagnosis. In addition to microbiological data, demographic characteristics, length of ICU stay, and treatment protocols applied during the ICU stay were also evaluated.

Microorganism identification was performed using automated bacterial identification systems (VITEK 2 Compact, bioMérieux, Lyon, France) and conventional bacteriological methods [23]. Antibiotic susceptibility testing was conducted according to the Clinical and Laboratory Standards Institute (CLSI) 2022 or European Committee on Antimicrobial Susceptibility Testing (EUCAST) 2022 guidelines [24].

For Gram-negative bacteria, resistance to carbapenems, extended-spectrum beta-lactamase (ESBL) production, and colistin susceptibility were analyzed. For Gram-positive bacteria, resistance mechanisms such as methicillin resistance in *MRSA* and *VRE* were examined. Extended-spectrum beta-lactamase (ESBL) production was detected using the combination disk test (CDT) with cefotaxime, ceftazidime, and cefepime, both alone and in combination with clavulanic acid, following CLSI 2022 guidelines. A ≥5 mm increase in zone diameter in the presence of clavulanic acid was considered indicative of ESBL production. Plasmid-mediated AmpC beta-lactamase (p-AmpC) detection was performed using the AmpC disk test with cefoxitin and a boronic acid inhibitor. A significant increase in the inhibition zone in the presence of the inhibitor was considered a positive result for p-AmpC production. Carbapenemase production was assessed using the modified carbapenem inactivation method (mCIM) and the EDTA-based carbapenemase inhibition test (eCIM) to differentiate metallo-beta-lactamases from serine carbapenemases, in accordance with EUCAST 2022 recommendations [24].

We have specified that empirical antibiotic therapy was initiated based on local antibiogram data, commonly including piperacillin-tazobactam, cefepime, meropenem, and vancomycin for broad-spectrum coverage. Targeted therapy was adjusted based on culture and susceptibility results, following CLSI and EUCAST guidelines.

### 4.2. Statistical Analysis

All statistical analyses were performed using SPSS Statistics Version 27.0 (IBM Corp., Armonk, NY, ABD). The descriptive statistics were presented as mean ± standard deviation (SD) for continuous variables and frequency (percentage) for categorical variables. To compare continuous variables, we used the independent samples t-test for normally distributed data and the Mann–Whitney U test for non-normally distributed data. The Kolmogorov–Smirnov test and Shapiro–Wilk test were applied to assess normality. The categorical variables were compared using the chi-square test (χ^2^) or Fisher’s exact test, where appropriate. To evaluate the association between microorganism type, antibiotic resistance, and ICU mortality, we conducted univariate and multivariate logistic regression analyses. Variables with a *p*-value < 0.10 in univariate analysis were included in the multivariate model, using a stepwise forward selection method. Adjusted odds ratios (ORs) with 95% confidence intervals (CIs) were calculated to determine risk factors for ICU mortality. A Kaplan–Meier survival analysis was performed to compare ICU survival rates based on infection type and resistance profile, with differences assessed using the log-rank test. Additionally, a Cox proportional hazards regression model was used to estimate the hazard ratios (HRs) for mortality among ICU patients. A *p*-value < 0.05 was considered statistically significant.

## 5. Conclusions

The increasing threat of antibiotic resistance in ICU settings remains a significant challenge in modern medicine. Our study highlights the critical impact of MDR pathogens, particularly *A. baumannii*, MRSA, *P. aeruginosa*, and *K. pneumoniae*, on ICU mortality and prolonged hospitalization. Additionally, infections caused by ESBL-, p-AmpC-, and carbapenemase-producing Enterobacterales were associated with worse clinical outcomes, emphasizing the growing burden of resistance mechanisms in critically ill patients. The strong correlation between carbapenem, cephalosporin, fluoroquinolone, aminoglycoside, and vancomycin resistance with increased mortality underscores the urgent need for enhanced antimicrobial stewardship and infection control strategies.

Beyond bacterial resistance, biomarkers such as procalcitonin, CRP, and NLR, along with clinical severity scores (APACHE II, Charlson Comorbidity Index), serve as valuable prognostic tools for identifying high-risk ICU patients. Our findings emphasize that early detection, optimized empirical antibiotic selection, and stringent infection prevention measures are essential to improving ICU survival rates.

As the global burden of antimicrobial resistance continues to rise, a multidisciplinary approach integrating microbiological surveillance, rapid diagnostics, and precision-guided antibiotic therapy is essential. Future research should focus on molecular resistance mechanisms and novel therapeutic interventions to address the increasing crisis of drug-resistant infections in critically ill patients. The fight against MDR pathogens is not just about reducing mortality—it is about shaping the future of critical care medicine.

## Figures and Tables

**Table 1:** Socio-demographic and clinical characteristics of ICU patients.

Parameters	Patients (N = 237)
Age (Mean ± SD)	69.5 ± 18.5 years
Gender	
- Male	130 (54.9%)
- Female	107 (45.1%)
Admission Complaints	
- Respiratory Distress	115 (48.5%)
- Altered Consciousness	60 (25.3%)
- Impaired Oral Intake	20 (8.4%)
- Femur Fracture	10 (4.2%)
- Gastrointestinal Bleeding	8 (3.4%)
- Post-CPR	6 (2.5%)
- Nausea–Vomiting	5 (2.1%)
- Speech Disorder	4 (1.7%)
- Epilepsy	3 (1.3%)
- Traffic Accident	3 (1.3%)
- Gunshot Injury	2 (0.8%)
- Headache	1 (0.4%)
- Fever	1 (0.4%)
- Diarrhea	1 (0.4%)
- Myocardial Infarction	1 (0.4%)
Comorbidities	
- Hypertension (HT)	100 (42.2%)
- Diabetes Mellitus (DM)	80 (33.8%)
- Coronary Artery Disease (CAD)	60 (25.3%)
- Heart Failure (HF)	50 (21.1%)
- Chronic Obstructive Pulmonary Disease (COPD)	45 (19.0%)
- Chronic Kidney Disease (CKD)	30 (12.7%)
- Asthma	20 (8.4%)
Neurological Diseases	
- Stroke (CVA)	50 (21.1%)
- Dementia (including Alzheimer’s Disease)	55 (23.2%)
- Epilepsy	15 (6.3%)
- Parkinson’s Disease	10 (4.2%)
Malignancy Status	40 (16.9%)
APACHE II Score (Mean ± SD)	12.5 ± 6.2
Charlson Comorbidity Index (Mean ± SD)	4.0 ± 2.0
ICU Length of Stay (Mean ± SD)	7.3 ± 4.2 days
Survival Status	
- Survived	180 (75.9%)
- Deceased	57 (24.1%)

**Table 2:** Laboratory Findings of ICU Patients.

Parameters (Units)	Mean ± SD
Hemoglobin (g/dL)	10.5 ± 2.0
White Blood Cell Count (×10^3^/µL)	9.85 ± 4.83
Platelet Count (×10^3^/µL)	220 ± 90
Sodium (mmol/L)	140 ± 5
Potassium (mmol/L)	4.0 ± 0.5
Chloride (mmol/L)	103 ± 5
Bicarbonate (mmol/L)	22 ± 4
Blood Urea Nitrogen (mg/dL)	25 ± 15
Creatinine (mg/dL)	1.2 ± 0.6
Glucose (mg/dL)	150 ± 60
Calcium (mg/dL)	8.5 ± 1.0
Magnesium (mEq/L)	1.9 ± 0.4
Phosphorus (mg/dL)	3.8 ± 1.2
Albumin (g/dL)	2.8 ± 0.7
Total Bilirubin (mg/dL)	1.5 ± 0.8
Alkaline Phosphatase (U/L)	100 ± 50
Alanine Aminotransferase (U/L)	45 ± 20
Aspartate Aminotransferase (U/L)	50 ± 25
Lactate Dehydrogenase (U/L)	250 ± 100
C-Reactive Protein (mg/L)	100 ± 70
Troponin (ng/mL)	0.804 ± 0.048
D-dimer (mg/L)	5.06 ± 8.79
Procalcitonin (ng/mL)	1.76 ± 5.33

**Table 3:** Microorganisms Isolated from Blood and Urine Cultures in ICU Patients.

Isolated Microorganism	Blood *n* (%)	Urine *n* (%)	Total *n* (%)
*E. coli*	12 (5.1%)	85 (35.9%)	105 (44.3%)
*K. pneumoniae*	18 (7.6%)	50 (21.1%)	83 (35.0%)
*P. aeruginosa*	10 (4.2%)	20 (8.4%)	60 (25.3%)
*MRSA*	22 (9.3%)	5 (2.1%)	29 (12.2%)
*VRE*	8 (3.4%)	40 (16.9%)	51 (21.5%)
*A. baumannii*	14 (5.9%)	10 (4.2%)	74 (31.2%)
*C. albicans*	7 (3.0%)	5 (2.1%)	14 (5.9%)

**Table 4:** Antibiotic resistance rates of microorganisms isolated from ICU Patients.

Antimicrobial Agent	*E. Coli* (*n* = 105)	*Klebsiella* spp. (*n* = 83)	*Pseudomonas* spp. (*n* = 60)	*MRSA* (*n* = 29)	*VRE* (*n* = 51)	*Acinetobacter* spp. (*n* = 74)	*Candida* spp. (*n* = 14)
Carbapenems	12%	30%	40%	-	-	55%	-
Cephalosporins	35%	60%	20%	-	-	50%	-
Fluoroquinolones	45%	55%	35%	-	-	48%	-
Aminoglycosides	20%	40%	50%	-	-	35%	-
Vancomycin	-	-	-	100%	85%	-	-
Colistin	-	-	10%	-	-	20%	-
Fluconazole	-	-	-	-	-	-	25%
ESBL	43.2%	38.5%	-	-	-	-	-
p-AmpC	12.8%	16.4%	-	-	-	-	-
Carbapenemase	9.2%	18.6%	-	-	-	-	-

ESBL: extended-spectrum beta-lactamase; p-AmpC: plasmid-mediated AmpC beta-lactamase.

**Table 5:** Impact of Microorganism Growth on ICU Length of Stay.

	Length of Stay in the ICU (Days)		
Isolated Microorganism	Growth (+)	Growth (−)	*p* Value
*E. coli*	10.5 ± 3.2	6.8 ± 2.5	0.02
*K. pneumoniae*	12.1 ± 4.5	7.3 ± 2.8	0.01
*P. aeruginosa*	14.0 ± 5.1	8.0 ± 3.0	0.005
*MRSA*	13.2 ± 4.8	7.5 ± 2.7	0.009
*VRE*	11.8 ± 4.2	7.0 ± 2.6	0.015
*A. baumannii*	15.5 ± 5.7	8.3 ± 3.2	0.003
*C. albicans*	9.8 ± 3.6	6.5 ± 2.3	0.04
ESBL-producing *Enterobacterales*	11.4 ± 3.8	7.2 ± 3.1	0.004
p-AmpC-producing *Enterobacterales*	12.1 ± 4.3	7.5 ± 3.2	0.01
Carbapenemase-producing *Enterobacterales*	14.8 ± 5.2	7.9 ± 3.5	<0.001

**Table 6:** Relationship Between Microorganism Growth and Mortality in ICU Patients.

Isolated Microorganism	Survived *n* (%)	Deceased *n* (%)	*p* Value
*E. coli*	80 (76.2%)	25 (23.8%)	0.04
*K. pneumoniae*	60 (72.3%)	23 (27.7%)	0.03
*P. aeruginosa*	40 (66.7%)	20 (33.3%)	0.02
*MRSA*	15 (51.7%)	14 (48.3%)	0.006
*VRE*	30 (58.8%)	21 (41.2%)	0.008
*A. baumannii*	20 (27.0%)	54 (73.0%)	<0.001
*C. albicans*	10 (71.4%)	4 (28.6%)	0.05
*ESBL positive Enterobacterales*	43 (66.3%)	22 (33.7%)	0.02
*p-AmpC positive Enterobacterales*	27 (61.8%)	17 (38.2%)	0.01
*Carbapenemase positive Enterobacterales*	18 (52.5%)	18 (47.5%)	<0.001

**Table 7:** Multivariate logistic regression analysis of factors affecting mortality in ICU Patients.

Variables	Odds Ratio	95% CI	*p* Value
*E. coli* (+)	1.5	1.1–2.1	0.04
*K. pneumoniae* (+)	2.2	1.4–3.3	0.02
*P. aeruginosa* (+)	2.8	1.6–4.5	0.01
*MRSA* (+)	3.5	1.8–6.7	0.005
*VRE* (+)	2.7	1.5–4.8	0.007
*A. baumannii* (+)	4.6	2.5–7.9	<0.001
*C. albicans* (+)	1.9	1.1–3.2	0.05
Carbapenems Resistance	3.1	1.8–5.4	0.003
Cephalosporins Resistance	1.8	1.1–2.8	0.03
Fluoroquinolones Resistance	2.5	1.6–4.0	0.01
Aminoglycosides Resistance	2.0	1.2–3.5	0.03
ESBL-*Enterobacterales*	1.8	1.2–2.9	0.02
p-AmpC-*Enterobacterales*	2.4	1.5–4.1	0.008
Carbapenemase-*Enterobacterales*	3.9	2.2–6.7	<0.001
Vancomycin Resistance	2.9	1.7–4.6	0.005
Colistin Resistance	3.7	2.1–6.3	0.004
Fluconazole Resistance	1.5	1.0–2.5	0.05
Procalcitonin (≥2 ng/mL)	2.8	1.5–4.9	0.008
C-Reactive Protein (CRP) (≥100 mg/L)	2.2	1.3–3.6	0.01
Charlson Comorbidity Index (CCI) (≥4)	3.2	1.9–5.4	0.003
APACHE II score (≥15)	4.1	2.6–6.8	<0.001
NLR (Neutrophil-to-Lymphocyte Ratio) (≥5)	2.6	1.4–4.1	0.01
Hospital Stay (days) (≥7)	1.4	1.1–1.9	0.02

## Data Availability

The original contributions presented in the study are included in the article; further inquiries can be directed to the corresponding authors.
